# A Rare Case of Plummer-Vinson Syndrome

**DOI:** 10.7759/cureus.6463

**Published:** 2019-12-25

**Authors:** Ajay K Gade, Lauren Pacheco

**Affiliations:** 1 Internal Medicine, Brookwood Baptist Medical Center, Birmingham, USA

**Keywords:** plummer vinson syndrome, iron deficiency anemia

## Abstract

Plummer-Vinson syndrome (PVS) is characterized by a triad of symptoms comprising microcytic hypochromic anemia, esophageal webs, and dysphagia. PVS is commonly found in women of middle age especially in the fourth and fifth decade of life and is rarely reported in males. We report a case of a 39-year-old female patient who had a classic presentation of PVS. PVS is precancerous with high malignant potential; early diagnosis is of utmost importance for better prognosis and surveillance endoscopy is recommended. Iron repletion oftentimes improves the dysphagia; seldom esophageal dilatation is used to provide symptomatic relief.

## Introduction

Plummer-Vinson syndrome (PVS) is a rare disorder associated with chronic severe iron deficiency anemia, leading to dysphagia, glossitis, and esophageal webs [1]. It is named after two American physicians Dr. Henry Stanley Plummer and Dr. Porter Paisley Vinson. PVS is also called Kelly-Paterson syndrome, named after two British otolaryngologists, Dr. Adam Brown-Kelly and Dr. Donald Ross Paterson [2]. On gross pathology, esophageal web and esophageal strictures are characteristic findings of PVS. On microscopic histopathological analysis, PVS presents with epithelial atrophy, chronic submucosal inflammation, and epithelial atypia or dysplasia in advanced cases [3-4]. Chronic irritation of the esophagus may predispose to an increased risk of developing esophageal webs or strictures. Common complications of PVS include hypopharyngeal cancer, esophageal cancer, and oral cancer [5]. Some differential diagnosis of PVS considered is dysphagia from achalasia, reflux esophagitis, esophageal carcinoma, esophageal spasm, systemic sclerosis, and Zenker's diverticulum [5]. Physical examination of patients with PVS is usually remarkable for pallor, glossitis, fatigue, and weakness [6]. Laboratory findings of PVS are consistent with the presence of iron deficiency anemia. Barium esophagogram is the best initial imaging study used for diagnosing PVS, which shows esophageal webs. Esophagogastroduodenoscopy (EGD) is also used to visualize esophageal webs and dilatation in a few cases [7]. The mainstay of treatment for PVS is aimed at correcting iron deficiency anemia.

## Case presentation

**Figure 1:**
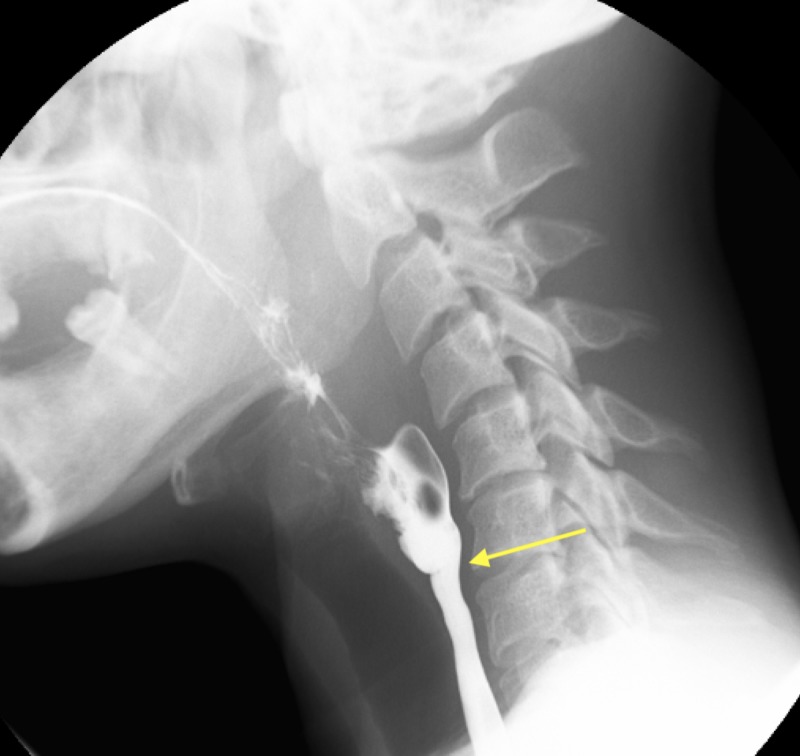
Barium swallow revealed a proximal esophageal web (arrow) with more than 50% luminal stenosis

A 39-year-old African-American female with no known past medical history presented to the emergency department (ED) with a sore throat, dyspnea on exertion, and substernal chest pain for three days. After being admitted for further evaluation, she mentioned having dysphagia with only solid foods prompting her to eat raw corn starch for the past 16 years, craving ice chips for the past six months, heavy bleeding with menses for years, and easy bleeding of her gums when brushing her teeth. On physical examination, her vitals are normal. She has conjunctival pallor, koilonychia, smooth tongue, and angular cheilitis. Labs were notable for hemoglobin/hematocrit of 6.3/25 with a mean corpuscular volume of 56.9, with a Mentzer’s index of 12.84, ferritin of 2, iron 81, total iron-binding capacity- 481. She was transfused 1 unit of packed red blood cells in the ED and later given parenteral dextran for her iron deficiency. To further workup her source of iron deficiency, her stool was negative for blood and pelvic ultrasound was notable for uterine fibroids. She also had hemoglobin electrophoresis which was unremarkable, although difficult to interpret in the setting of a recent blood transfusion. To further workup her dysphagia, she underwent a barium swallow which was notable for a proximal esophageal web with more than 50% luminal stenosis (Figure 1). Gastroenterology was consulted and she underwent EGD with esophageal dilatation which completely resolved her dysphagia with solids by discharge.

## Discussion

PVS was suspected in this patient with iron deficiency anemia and dysphagia; it was then confirmed after her barium swallow. The syndrome is a rare disorder characterized by a triad of iron-deficiency anemia, dysphagia, and esophageal webs [8]. The exact pathogenesis of PVS is unknown. It is postulated that PVS results from iron deficiency [9]. Other possible factors include malnutrition, genetic predisposition, and autoimmune disorders. In patients with iron deficiency, the iron-dependent oxidative enzymes are unable to function at an optimum level and the dependent metabolic pathways such as oxidative phosphorylation are compromised. This promotes anaerobic metabolism leading to myasthenic changes in esophageal muscles, which in turn forms esophageal webs. The dysphagia is rarely due to esophageal muscular discoordination [10-11]. Patients with iron deficiency have low levels of myoglobin which may affect the muscles of the tongue and lead to glossitis. Genes involved in the pathogenesis of iron deficiency anemia associated with PVS include a mutation in the TMPRSS6 gene [12-13]. The TMPRSS6 gene encodes instructions for the protein hepcidin. Increased levels of hepcidin lead to decreased release of iron from ferritin and subsequently presents as iron deficiency anemia [14]. Esophageal webs and esophageal strictures are characteristic findings of PVS on the gross pathology. Microscopy can be notable for epithelial atrophy, chronic submucosal inflammation, and epithelial atypia or dysplasia in advanced cases. Medical therapy involves replacing the iron stores which can decrease the dysphagia symptoms effectively. Surgery is not the routinely recommended treatment option for patients with PVS [15-16]. In case of significant esophageal obstruction by multiple esophageal webs or persistent dysphagia despite medical treatment, rupture and mechanical dilation of the web using an endoscope can be performed such as with our patient [16].

## Conclusions

It is important to further evaluate any complaint of dysphagia and to be cautious of any red flag warning signs. Especially in the setting of glossitis on the exam and severe iron deficiency anemia on labs, consider evaluating for esophageal webs as the etiology of dysphagia. Managing the esophageal webs of PVS and repleting iron stores at an early stage is crucial as the webs can progress to esophageal or pharyngeal squamous cell carcinoma.
